# Institutions of Care: A Qualitative Study with Ancestral Black Nova Scotian Nurses in Healthcare

**DOI:** 10.1177/08445621241313421

**Published:** 2025-01-28

**Authors:** Keisha Jefferies, Ruth Martin-Misener, Gail Tomblin Murphy, Jacqueline Gahagan, Wanda Thomas Bernard

**Affiliations:** 1 School of Nursing, Faculty of Health, 3688 Dalhousie University, Nova Scotia, Canada; 2 Research, Innovation and Discovery, 432234 Nova Scotia Health, Nova Scotia, Canada; 3 Research Office, 3684 Mount Saint Vincent University, Nova Scotia, Canada; 4 School of Social Work, Faculty of Health, 3688 Dalhousie University, Nova Scotia, Canada

**Keywords:** Ancestry, Black/African American, healthcare, integration, nursing practice, professional nursing / nursing education / nursing administration

## Abstract

**Background:**

Ancestral Black Nova Scotian (ABNS) nurses are a culturally distinct group yet, little is known about their experiences. Available literature suggests that ABNS nurses are underrepresented in nursing and that they encounter discrimination throughout the health system. Understanding the experiences of ABNS nurses facilitates addressing antiBlack racism in nursing and healthcare.

**Purpose:**

This study sought to critically examine the leadership experiences of ABNS nurses in healthcare.

**Methods:**

This qualitative study was guided by Black feminist theory and involved one-on-one semi-structured telephone interviews with eighteen ABNS nurses. Critical Discourse Analysis was applied in the reading of interview transcripts to examine words used by participants in relation to nursing and healthcare. The findings are presented in two conceptual themes.

**Results:**

Black Tax in Nursing captures the added physical, mental, and spiritual strain experienced by ABNS nurses navigating nursing and healthcare. Black Tax encompassed everyday microaggressions and systemic processes, including intra-profession tensions. Integrating into nursing was made increasingly difficult by a reinforcing network of gatekeepers, policies, and structural design. Nova Scotia Healthcare as an Archaic Institution depicts an antiquated “broken” paternalistic system that did not empower patients nor promote health. Additionally, nursing education was accused of reinforcing negative stereotypes, competency gaps, and mistrust with patients.

**Conclusions:**

Institution of Care show how ABNS nurses challenge institutional standards and norms in their approach to nursing. ABNS nurses navigate nursing and the health system by maintaining a community-oriented approach to health. Addressing anti-Black racism in nursing and healthcare requires attention to multi-level processes within institutions.

Ancestral Black Nova Scotian (ABNS) nurses have an extensive history of informal caring in their family and communities. Yet, due to historical and present-day systemic, institutional, and social barriers, ABNS are underrepresented in the nursing profession and throughout healthcare. This qualitative study was guided by Black feminist theory and critically examined the leadership experiences of ABNS nurses in healthcare practice. As a distinct group of people, within the larger population of people of African descent (PAD) in Nova Scotia, the experiences of ABNS nurses are either aggregate within the broader PAD experience or excluded entirely. This research addresses the paucity of evidence regarding ABNS nurses, describes their experiences in healthcare, and presents recommendations for advancement.

## Background and purpose

Ancestral Black Nova Scotians (ABNSs) are a culturally distinct group, with ancestral land-based connections, within the larger population of PAD in Nova Scotia. ABNS heritage, dating back to the 1600 s, has resulted in a uniquely complex experience of Blackness (African Nova Scotian Strategy Advisory Council, 2021; Jefferies, 2024; Pachai, 1997; Whitfield, 2018). For example, many socioeconomic issues encountered by ABNSs are the result of centuries of anti-Black racism in Nova Scotia (United Nations [UN], 2017). Anti-Black racism is a form of racial discrimination experienced by PAD, where bias and prejudice manifests in policies, systemic processes, and institutions (Black Health Alliance [BHA], 2018). Anti-Black racism permeates multiple sectors of society and significantly impacts PAD in Nova Scotia and Canada by exacerbating disparities throughout education, health, employment, and housing (Jean-Pierre, 2021; Kisely et al., 2008; UN, 2017). Further, disparities in educational attainment and access to higher education for ABNSs are apparent when compared to the general Nova Scotia population as well as the larger PAD population in Nova Scotia.

The impact of anti-Black racism on the health, wellbeing, and overall nursing practice continues to be uncovered (Beagan et al., 2023; Cooper Brathwaite et al., 2022; Das Gupta, 1996, 2009; Jefferies et al., 2022a). Premji and Etowa (2014) found that Black nurses were underrepresented in nursing, particularly in specialty care areas and in advanced practice and leadership. Additionally, Black nurses report encountering interpersonal and institutional barriers related to career advancement (Boateng & Adams, 2016; Turrittin et al., 2002). Many of these contemporary challenges are linked to policies that reinforce anti-Black racism in nursing, including restricting access and prohibiting Black women from practicing nursing (Calliste, 1993; Flynn, 2011).

Significant evidence gaps related to the experiences of ABNS nurses in healthcare and leadership reinforce, and are reinforced by, structural and systemic barriers (Beagan et al., 2023; Etowa et al., 2009; Jefferies et al., 2022a; Jefferies & Price, 2021). The continued failure to acknowledge and address anti-Black racism in nursing and healthcare in Canada has increased health disparities, inequities, as well as reduced our collective knowledge regarding PAD in Canada (Canadian Nurses Association [CNA], 2020, 2021). Further, existing literature regarding Black nurses in Canada focuses largely on the experiences of Black immigrant nurses, in larger more diverse metropolitan regions such as Ontario or British Columbia, Canada (Jefferies et al., 2022b; Kihika, 2024). This literature informs a fulsome understanding of issues pertaining to the collective of Black nurses in Canada. However, it fails to account for the heterogeneity that exists among PAD in Canada, especially when gaps in education and career advancement are considered. There is limited research focused on ABNS nurses (Chaplan, 2020; Etowa et al., 2009; Flynn, 2011; Keddy, 1997) with a further paucity of evidence on the impact of ancestry or generational status (Jefferies, 2024). To address these long-standing evidence gaps and to truly inform nursing and health system transformation, research must examine the multitude of factors, including ancestry, gender, disability, geographical location, generational status, class, and how PAD in Canada are impacted (Cénat, 2022).

As an ABNS nurse with 10 years of nursing experience, I encountered many of the challenges reported in the literature (Jefferies & Price, 2021). Additionally, I witnessed myriad challenges for ABNS nurses that are not captured in the literature. To this end, the purpose of this qualitative study was to critically examine the leadership experiences of ABNS nurses in healthcare. This study sought to answer two research questions: 1) What are the leadership experiences of ABNS nurses? and 2) How do ABNS nurses perceive leadership? This paper presents findings from Section Two of a larger qualitative study that examined the leadership experiences of ABNS nurses in healthcare (Jefferies et al., 2022a).

The legacy of anti-Black in nursing has limited advancement in the profession. Acknowledging these historical transgressions, actively addressing anti-Black racism, and ensuring the representation and inclusion of historically excluded groups is vital for institutional and systemic transformation. These findings hold significance for ABNS nurses, the wider Canadian health system, and population health. Additionally, these findings challenge traditional norms, standards, and assumptions in nursing and healthcare. Finally, by illuminating gaps in nursing and the health system, these findings offer recommendations for institutions to address barriers encountered by ABNS nurses in nursing education and in the health system.

## Methods and procedures

This qualitative study was guided by Black Feminist Theory (BFT), which is a critical social approach to research that examines how social, structural, and institutional systems reinforce oppression and impact experience (Barbee, 1994; Collins, 1990; Davis, 1981; De Sousa & Varcoe, 2022; Lorde, 1984). BFT was selected as the theoretical framework for this study for three main reasons. First, BFT unapologetically centers the ideas, interpretations, and experiences of Black women. Second, BFT emphasizes the significance of historical and social context. Third, BFT maintains the importance of oral history and dialogue in understanding experience. The study setting was a province in Eastern Canada. The population of interest was ABNS nurses. The study sample included participants identified as 1) a nurse and 2) of ABNS ancestry. Participants across all nursing designations, in all areas of nursing practice – clinical, policy, education, research, and administration – were eligible to participate, including retired nurses. Importantly, a formal leadership title was not required for eligibility. For ABNS ancestry, participants were eligible if they had at least one Black parent who was born and raised in Nova Scotia. This study did not impose restrictions related to the sex or gender of participants. Participants were excluded from the study if they were not a nurse and were not of ABNS ancestry. Examples of excluded populations include nursing students, non-licensed care providers, other health practitioners, and PAD who did not have one Black parent who was born in Nova Scotia.

Participant recruitment and data collection occurred concurrently from January 2020 to June 2020, using purposive and snowball sampling to recruit and select participants who could meaningfully inform understanding the leadership experiences of ABNS nurses in healthcare (Jefferies et al., 2022a; Sandelowski, 1995). Recruitment materials, distributed through personal and professional networks, are appended in published work (Jefferies et al., 2022a). Data were collected through one-on-one telephone interviews using a semi-structured interview guide. The interview guide was developed by the first author then reviewed and approved by a doctoral committee with expertise in the areas of ABNS care providers, qualitative research, intersectionality, and nursing workforce. The interview guide, which is appended as a supplemental file in published work (Jefferies et al., 2022a), was also approved by two independent Research Ethics Boards. Importantly, the interview guide did not collect demographic data and the guide was not shared with participants prior to the interview.

Consent to participate in the study and to have the interview audio-recorded, was obtained from each participant. Participants were informed of the option to withdraw from the study up to two-weeks after the interview. Each participant was offered a $30 electronic money transfer as a token of appreciation for participation in the study. Interviews were conducted by the first author, lasted 30–90 min in duration, were audio recorded and then transcribed by a professional transcriptionist. Interview transcripts were cleaned and organized by the first author. Trustworthiness involved attending to credibility, confirmability, dependability and transferability, using peer-debriefing, an audit trail, journaling and memoing (Creswell, 2013; Lincoln & Guba, 1985; Jefferies et al., 2022a).

Data collection continued until no new participants expressed interest in participating in the study. This approach to recruitment and data collection was a methodological decision made by the first author and aligns with the tenets of BFT. This decision prioritized the importance of storytelling, oral history, and the continued exclusion of ABNS experiences from the literature. The first author ensured that any ABNS nurse who wanted to share their experience received the opportunity to do so. Additionally, this study did not collect any demographic data. Demographic data, such as age, gender, sex, ABNS home community, as well as nursing designation, were not collected. The intentional omission of these demographic data was done to protect the privacy and confidentiality of the participants. The ABNS population comprises a small percentage of the Nova Scotian population and a smaller percentage of the nursing workforce. Reporting demographic data in relation to this population would increase the likelihood of identification of the participants.

Data analysis was an iterative process, involving the reading and rereading of interview transcripts. As described in Jefferies et al., 2022a, Critical Discourse Analysis (CDA) (van Dijk, 1993, 1995, 2015) is a critical approach to research that analyzes language in the form of talk and text. In this study, CDA was used to understand and describe participants’ experiences, across three levels – the discursive, social, and cognitive. Together, the analytical framework of BFT and CDA facilitated the examination of the words and phrases used by participants to describe experiences in nursing and healthcare. This included highlighting the terms and phrases (CDA) used by participants while describing the meaning alongside the historical and social context (BFT). Further, examining participants’ words and phrases elucidated the meaning ascribed to leadership by ABNS nurses. Additional details related to the analysis, including a description of each level of analysis is included in Jefferies et al., 2022a. Data were then classified into overarching conceptual categories, generative themes and subthemes (Jefferies et al., 2022a).

## Results

This paper presents findings from *Section Two* of a larger qualitative study (Jefferies et al., 2022a)*,* with ABNS nurses. The study database consisted of interview transcripts from 18 ABNS nurses. A non-descript overview of demographics include participants representing various ABNS communities across the province, diversity in age and sex, representation across each nursing designation, varying seniority, multiple practice areas, and many completing their nursing education at one of the nursing programs in the province.

**Figure 1.**
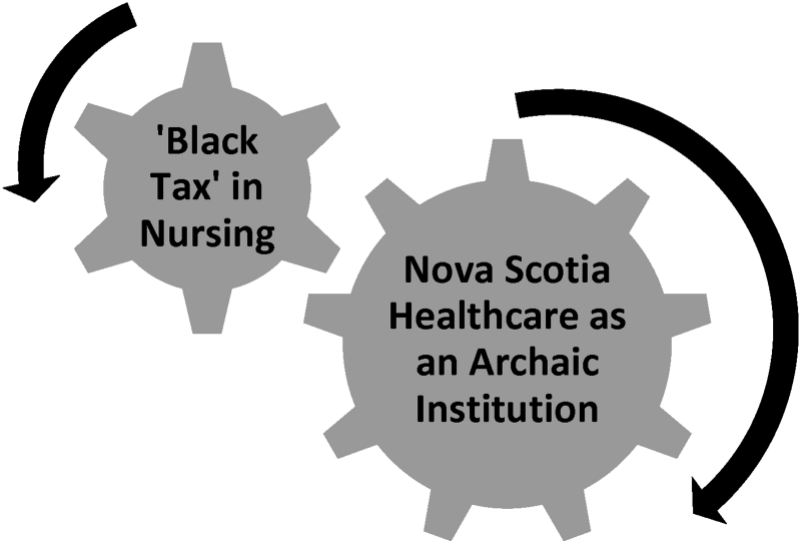
Section two: institutions of care overarching themes.

The two institutions, the nursing profession and the healthcare system, are presented as two themes, ‘*Black Tax’ in Nursing* and *Nova Scotia Healthcare as an Archaic Institution*, illustrated in Figure 1.

### Theme 1: ‘Black Tax’ in nursing

**‘**Black tax’ in nursing conceptualizes the added mental, physical, emotional, and spiritual strain that participants experienced as they navigated the nursing profession. Black tax exists in environments, where racial hierarchies are present, and manifest as an omnipresent and insidious undertone. Notably, Black tax is amplified by intersections of gender, class, sexual orientation, and disability.

“It is a grueling world… You have to remember the procedures and what you have to do. But at the same time, you also have to protect yourself” (R#5).

The theme, Black tax in nursing, is presented in four subthemes*: 1) Nursing as a Service: The Blending of Art and Science 2) Nursing Politics: Navigating Intra-Professional Tensions 3) Nursing Education: Primer for Praxis* and *4) Invite Only!: Gatekeepers, Standards and Structural Design.*

#### Subtheme 1: nursing as a service: the blending of art and science

Participants described nursing as a dynamic service-based profession, with a multitude of possibilities for career trajectory and fulfilment. Nursing was described as a profession requiring critical thinking, setting aside ego, and providing holistic person-centred care. Participants acknowledged heavy workloads, increasingly complex patients, and the risk of falling into patterns of tasks or administrative work. Yet, despite the demands of busy units, and collegial tensions from conflicting beliefs about *nursing as a service*, participants strived to maintain person-centered care. Still, the evolution of the profession challenged person-centered care and the art of nursing. For example, supporting activities-of-daily-living was viewed as central to nursing and a way to maintain person-centeredness.

It's a service industry… it's cleaning up blood, body fluids, feces. People are at their worst when they're sick. Maybe they're nice when they're well, but they're not when they're sick. I've had people throw trays at me. I don't think it had anything to do with my colour. I think it had to do with the situation they were in…So why spend four years training for something that is a service that's sometimes appreciated and sometimes not? You have to love nursing to really appreciate it…It's not an easy job… Nursing is very intimate. (R#2)

Participants shared how some units and policies stifled person-centered care. Yet, participants strived to attend to the social and promotional dimensions of health, including caring for the physical, mental, and spiritual needs of patients.

I wanted to stay within mental health. A lot of the nursing roles that I had had didn't have that traditional nursing feel. It was looking at other ways to increase well-being. My role focused on that social piece, the wellness piece, the engagement piece. That was something that I really enjoyed. (R#9)

*Nursing as a service* extended to include families and community. Participants welcomed and encouraged family involvement and participation in care, which they believed increased trust, patient satisfaction, and a greater understanding of health issues. On occasion, the desire to include families conflicted with that of colleagues.

I have more sensitivity towards people. I don't jump to conclusions. I always consider that there's a reason as to why something's happening before I assume the worst of a person… And I'm big on family-centred care, the family being involved. I find a lot of nurses complain about the family. (R#14)

*Nursing as a service* integrated the art and science of the profession. Additionally, childhood experiences, including witnessing caregiving, contributed to this belief and fuelled the desire to provide the best care. Ultimately, many participants expressed that those who enjoyed nursing had a passion for service to others.

#### Subtheme 2: nursing politics: navigating intra-professional tensions

Participants described navigating common intra-professional tensions related to seniority, RN-LPN dynamics, and the balance of theory and practice. One participant used the term “jaded” to describe nurses nearing retirement, who did not support junior nurses. While other participants felt that they were given a hard time, needed to prove themselves, or modify their behaviour or speech (code-switch), due to age- or race-based assumptions.

Because I look younger than I am, they treat me like I don't know what's going on. Initially they act as though I know nothing… I find that happens a lot where I feel I have to prove that I'm just as good as everyone else… It really depends on who I'm working with. A lot of the time I feel I have to talk a certain way to get respect. If I don't code switch, people automatically dismiss me. If I don't talk white, what people say is white. (R#14)

Another intra-professional tension was the RN-LPN dynamic. Participants challenged assumptions related to knowledge, critical thinking, and leadership. Participants expressed appreciation for the advanced skill level of LPNs, who were described as valued team members. Some participants felt that emphasis should be placed on competence, rather than defaulting to title or scope. This perspective was paired with the belief that quality care transcended titles or institutional restrictions.

On that unit, it's RNs only. Because it's acute they tried to say the LPNs don't have the qualifications. However, LPNs are doing more. And I would stick up for them because it's like you are giving the heavy loads to the LPNs when you’re all RNs. (R#16).

Building on the RN-LPN dynamic, a related tension involved the balance of theory and practice. Several participants remarked on the importance of possessing theoretical knowledge and practical skills. Though, concern was expressed with the increasing emphasis on theoretical knowledge, and less on practical skills. Importantly, the shift from the practical to theoretical was a suspected contributor to diminished care, particularly in the acute care setting.

“I find LPNs are more prepared. They have more hands-on experience than the nurses coming from baccalaureate programs. The RNs coming in have more book smarts. But with procedures, LPNs are bang on their stuff” (R#12).

Another participant explained:

You need the theory. But there needs to be more practical hands-on learning for nurses…Just the basics. I know that the healthcare system is struggling because there aren't enough staff. But… even though we dealt with very heavy patients, we had large workloads, we still tried to maintain a degree of I'm here to help you. We encouraged people to do what they could for themselves. But if they couldn't, we tried to accommodate… it's important that students have hands-on clinical experience, community, and community hospital…Even though people are stressed and it's difficult, there has to be a level of professionalism that helps us stay committed to patient care. (R#6)

The theory and practice tension raised questions about the valuation of practical skills and experience in nursing, which was evident in comparisons of knowledge and skills between senior nurses and new graduate nurses.

I would have been practicing for 25 years. And my boss told me that a new graduate would have as much knowledge and experience as I would have. I must say, I smiled and thought big R was in the room there. (R#5)

Participants were critical of the status quo and assumption-based practice, while acknowledging intra-professional tensions. The complexity of these dynamics was apparent, with participants describing tensions with senior nurses while also sharing interactions with senior nurses who were helpful and fostered a supportive learning environment. Finally, the shifts in nursing were acknowledged as occurring within a larger health system, that required changes to address the growing complexity of patient and community needs.

#### Subtheme 3: nursing education: primed for praxis

Nursing education became a primer for praxis – including leadership, critical engagement, and advocacy. Participants challenging and disagreeable situations including being only one of two or three Black students in programs with more than 100 students. The impact of being *the only one* was compounded by being a mature student, a working parent, visibly Black, and a first-generation university student.

There were just a few of us in a room of 130 people… it was very intimidating… I don't have any nurses, doctors or anybody that's done a science degree in my family. Who am I going to talk to about this? …But it was not only being in an environment that I didn't feel comfortable or accepted in, there was nobody gravitating towards me. Plus, I'm a mature student. I'm leaving school and going to work. I have children. I felt outside like, I don't fit in…So it wasn't easy… At times, I was like how am I going to do this, how am I going to get through it? But it was my friends that I came through [program] with that kept me grounded because we would meet up and go places. (R#3)

Participants also remarked on gaps in nursing education, including the limited number of courses that integrated diversity, the social determinants of health, and additional content related to racially and/or ethnically diverse group in Canada. This forced participants into teaching classmates and professors about these topics.

Just one course in the whole four years. It was trying to cover a lot in terms of social determinants of health. I don't know what the curriculum is like now, but there needs to be greater inclusion of diversity, of race, of religion, of, gender identity. More relational things. (R#11)

Another participant expounded:

The course was us doing a presentation on a group of people. They should be teaching about Black and Indigenous people. Yes, of course they teach about white people. But you need to teach about who else is here. There's more Asian people, there's Syrian people. (R#16)

Clinical placements were another challenging experience, with participants describing being singled out, based on appearance. As student learners, participants were forced to educate patients on racism and the inappropriate nature of their derogatory terms and racist comments, during their clinical placement.

I remember one of my first clinicals was at a nursing home. A lot of the residents had dementia… some patients would call me “the coloured girl”. It made me feel I was sticking out. I'm a student like everyone else. Why is it that I have to be identified by my appearance? I'm doing everything that I should be doing. But I guess it's just with the times. Now, it doesn't bother me as much as it did back then. (R#7)

Another illustrative quotation:

You feel like you're all alone. I have no one that looks like me, no one that can relate to me. You encounter things just being visibly Black. It's a given, especially in older populations. I’ll never forget the first time I had a patient that said ‘the n-word’. I was a student… He didn’t say it to me but he said it in conversation to refer to Black people. I was really assertive, and I was like, you can't use that word. It's a derogatory term towards people that look like me. It's very offensive. That's not acceptable. He was an older man and he was like, “I didn't mean it like that. (R#10)

Nursing school provided a unique set of circumstances, beyond the program requirements, that were a primer for praxis. Participants developed a sharp and eloquent way to critically assess and respond to situations, as well as advocate for themselves and others. Participants also took on the role of educating classmates and professors about the experiences of PAD in Canada, describing the significance of the social determinants of health, and defending equity policies and initiatives.

#### Subtheme 4: invite only!: gatekeepers, policies and structural design

**Figure 2.**
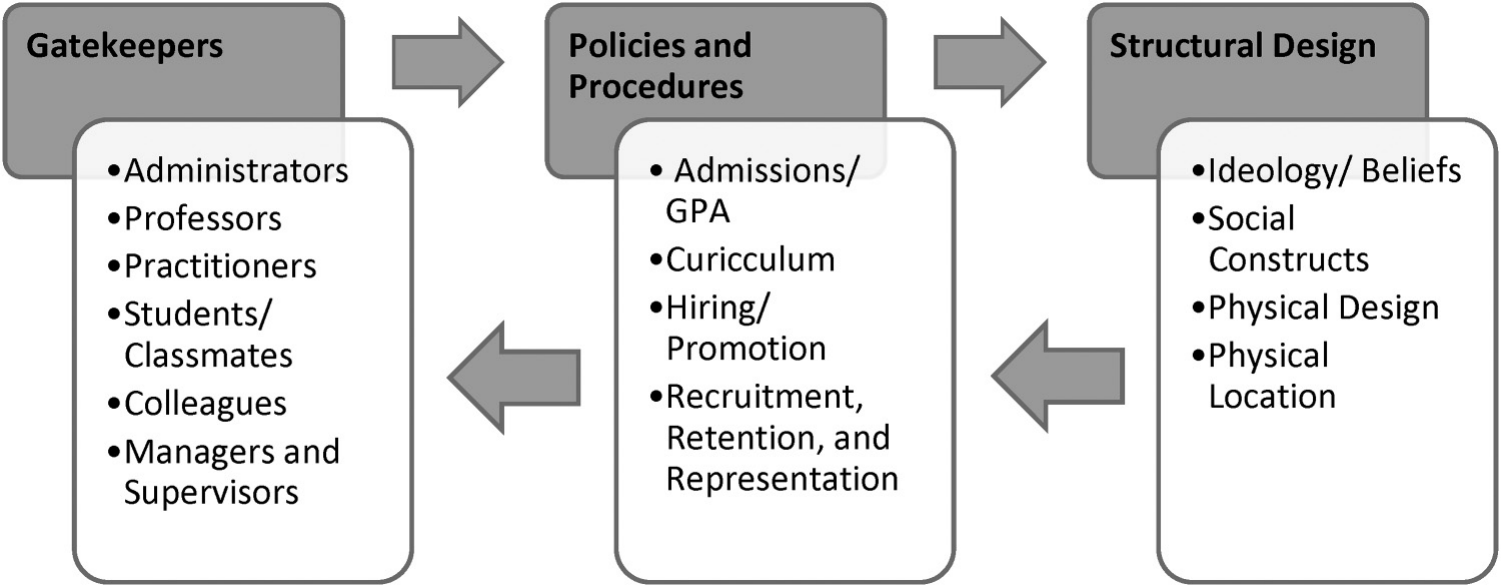
Process of integration into institutions.

The fourth subtheme describes the insulation of the nursing profession as an exclusive club. This bi-directional process is conceptualized in an integration model, illustrated in Figure 2. The process was present in nursing education and practice, and includes three levels: *gatekeepers, policies*, and *structural design*.

The first level, *gatekeepers*, depicts individuals who utilize institutional policies and procedures to uphold and reinforce institutional ideology. Gatekeepers, often the initial contact person, were described as intentionally withholding crucial information and operationalizing policies and procedures in a discretionary [often discriminatory] manner, to insulate the profession by filtering out less desirable or unsuitable prospects, to maintain an antiquated sense of purity in nursing. Some participants described how administrators were dismissive of participants’ inquiries and occasionally misled or misdirected participants. The withholding of pertinent information, such as the status of an application, or discussing program information in an assumptive manner about the ability of participants to gain admissions or complete the program, were shared.

In practice, the gatekeeper gaze included overlooking for promotion and excessive monitoring. Participants described being passed over for promotion despite extensive experience and qualifications. Nursing as an “old girl's club” reinforced the idea that nursing is an exclusive club reserved for desirable members who are scrutinized for suitability beyond nursing capabilities. While surveillance and disproportionate scrutinization were additional tactics used by gatekeepers.

The participant shared:

I was told that you don’t answer the call bells. But you can disregard that because I watched you all morning, and you don't stop.” I'm thinking, well, if you watched me all morning, why are you bringing this to my attention? Go back to whoever told you this blatant lie and correct them. (R#8)

The second level included the *policies* and processes that are constructed from the ideological design and operationalized by gatekeepers. Participants described how policies and procedures, such as hiring or admissions, were not inclusive, and served to further restrict access to nursing. Additionally, participants highlighted the discrepancy between admissions requirements for the nursing program and the qualities that contribute to competency and success as a nurse.

Beyond the use of policies and procedures to insulate the profession, participants described how a lack of transparency and sufficient channels made the process of filing complaints or grievances and sharing concerns challenging.

“I got pushed and pulled around. It wasn’t the supportive backbone that I was expecting. You’re only given a year… bring it to them. So if [they] bypass that year because you're doing meetings, how can you bring a claim?” (R#18).

Level three included the *structural* or institutional design, which involved aspects such as the ideological scaffolding and the physical structure or location. For example, participants explained the importance of fostering environments where Black students and nurses feel welcomed, supported, and a sense of belonging.

There needs to be a place where Black nursing students feel comfortable talking about their concerns without being penalized. I know people who have dropped out of the program and they didn't feel that they had anywhere to go with their concerns. (R#6)

Another participant continued:

“I have Black nurse colleagues at work who I connect with. We get together for lunch or go for a walk on a break. You need a space where you're not the only one who looks like you” (R#13).

The integration process involved gatekeepers reinforcing policies, which emerged from structural design. Thought another way, structures prescribed policies, which in turn were operationalized by gatekeepers. Integrating into nursing was challenging and efforts to integrate did not always result in acceptance into the exclusive club. This process forced a hyper-self-awareness, with participants questioning how they were perceived. Notably, many participants attributed racism as a substantial barrier to integration. While others described how the interplay of gender, age, and class created additional barriers to integration. Finally, a diminished sense of belonging combined with daily microaggression was exhausting.

### Theme 2: Nova Scotia healthcare as an archaic institution

The Nova Scotia healthcare system was compared to healthcare systems in other provinces and countries, which were considered more advanced in terms of patient care, diversity among practitioners, and health perspectives. Gaps in the Nova Scotia system were identified by participants who practiced in other jurisdictions as well as those who practiced predominantly in Nova Scotia.

“Nova Scotia is backwards and they need to come to grips with the fact that the world is changing around them” (R#4).

“Nova Scotia doesn't really promote educational and leadership opportunities. Whereas in [province], there were always conferences, workshops, and funding with the hospital and the union. I don't find those in Nova Scotia” (R#1).

“Some things have gotten better but there's still not as much as there should be. Nova Scotia is a small place. So you have more opportunity” (R#2).

**Figure 3.**
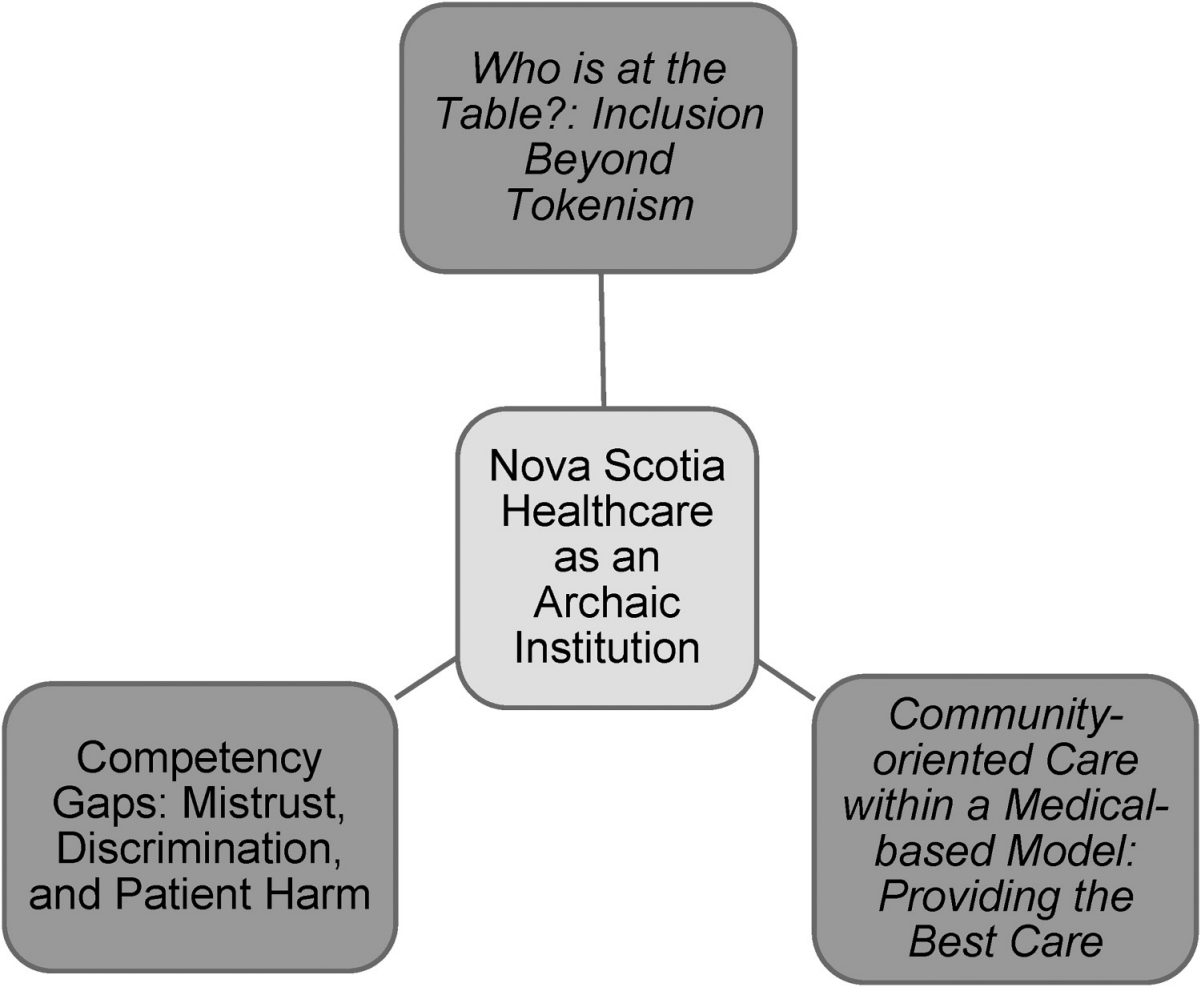
Nova Scotia healthcare as an archaic institution.

The theme, Nova Scotia healthcare as an archaic institution, contains three subthemes *1) Who is at the Table?: Inclusion Beyond Tokenism; 2) Competency Gaps: Mistrust, Discrimination, and Patient Harm; 3) Community-oriented Care within a Medical-based Model: Providing the Best Care*, illustrated in Figure 3.

#### Subtheme 1: who is at the table?: inclusion beyond tokenism

Inclusion beyond tokenism conceptualizes diverse representation of practitioners and perspectives beyond tokenism. Diversity was considered an essential component for care, whereas tokenism and diversity quotas resulted in feelings of being used by organizations and institutions. Further, being the only Black person was difficult and lonely, with some participants not wanting to “sit at the table” alone. Sitting at the table alone left some participants feeling ignored or undervalued. Yet, the importance of visibly diverse representation in healthcare was viewed as beneficial to collegiality and comfort and trust for patients. Participants also shared how diversity has the potential to improve health and address community-specific needs by incorporating relevant information into directives, policies, and teaching.

We bring another perspective. When I started working here, there wasn't any testing for sickle cell anemia. We meet regularly here to talk about things that need to be implemented. And one thing we talked about was having screening for sickle cell. Now we screen all prenatal patients…We can see things through a different lens when we have Black healthcare professionals bringing a perspective that wasn't there before. (R#6)

Omission of community-specific content ranged from the failure to acknowledge the unique experience of ABNSs to the exclusion of basic information on the history of ABNSs. Participants found attempts to incorporate community-specific information as performative as opposed to a genuine investment and interest in community. Participants also contrasted their experience with other groups who have been historically marginalized in Nova Scotia; expressing frustration that despite the presence of ABNSs in Nova Scotia for over 400 years, little was known. While, the information that was included, was described as inaccurate, false, or prejudicial. To rectify this, participants felt that ABNSs should be actively recruited into nursing and that the community should be involved in policy and program development.

I've always been super aware that there's no one else that looks like me. That's an apparent thing in my unit. Have I had negative experiences related to that? No. But it is… sad to me that there isn't more. Because again, growing up, what was important to me was seeing myself reflected in society… I had never seen that in nursing. (R#11)

Racist comments from patients were suspected as stemming from the lack of diversity in healthcare. Many racist encounters involved older patients, those in psychosis, or those with cognitive degenerative disorders such as dementia. Participants also highlighted generational differences and how racist rhetoric, towards Black people, was more acceptable during previous eras.

“I’ve been called everything. I thought my name was bitch once. I’ve been called out of my name a few times” (R#2).

“I get so much stuff dealing with the elderly. And it's always the elderly of course” (R#15).

When you have a few white patients in psychosis, it's “Black this and Black that.” I’d just laugh because they don't know what they’re saying. I'm like that doesn't hurt me. That doesn't bother me. They would think that that's affecting me. But nah. (R#16)

Some participants described little follow up after racist encounters with patients or colleagues. On occasion, colleagues would suggest that comments were not offensive, that they were a joke, or it was not a big deal. These responses and lack of follow up, diminished the sense of belonging.

I've been asked to not come into patients’ rooms because I don't look like anyone they've seen. I've been told I don't speak properly, and they would not like to see me for the rest of the shift. Then when I ask to be switched, it's humming and hawing. Meanwhile I'm fighting back tears. (R#18)

#### Subtheme 2: competency gaps: mistrust, discrimination, and patient harm

Participants explained how competency gaps stemming from education in paternalistic, disconnected programs led to mistrust, discrimination, and patient harm. Participants emphasized the need for culturally informed education and training, working collaboratively with the community, as well as evidence-informed policies and directives with evidence generated within and by the community.

“There's history that you may not be aware of and interactions with this community coming into the hospital because you're not from this province” (R#18).

Knowledge and service gaps were thought to disproportionately affect populations that have been historically marginalized. Participants explained how poor treatment from practitioners reinforced mistrust, whereas cultural considerations and an understanding of life circumstance were thought to improve trust and care. For example, participants described advocating for appropriate products to care for hair and skin.

We used Alco gel. That was pure alcohol. It's bad for anybody's skin. But for Black skin, it was horrendous. I spoke with our manager about having a different kind of [lotion]. Vaseline Intensive Care at least?… I was happy when I would get Black patients because I could care for their skin and hair properly. But it was also a statement about the staff and the nurse managers… I had to address that… I appreciate you assigning me patients of African descent but I think other people should be assigned as well. (R#6)

Participants described additional dangers of assumption-based care, prejudice, stereotypes, and a lack of cultural competency and how these had severe implications such as the removal of children from families. Shortcomings in professional education, and a lack of culturally competent educators, were cited as sources of competency gaps. Further, several participants accused nursing education as reinforcing harmful stereotypes and inadequately preparing nurses to care for deeper causes of issues for diverse populations.

“I don't see any Black content as far as health assessments with a Black person in the curriculum…There's no Black education in nursing. There never has been. I don't know if there ever will be” (R#4).

Other participants expressed concerns with simulations, where it was felt that simulations reinforced false and inaccurate information related to Black people, which was considered detrimental to the nurse-patient relationship.

They tried to incorporate different ethnicities but it was more stereotypical. I remember we had this online simulation and it was a Black lady. And just different characteristics that she had that white people don't have. Like hair on her upper lip or her elbows are darker. Certain things that people don't understand and weren’t talked about. Or when you wash a Black person's skin, the facecloth often gets dark because their skin is dark and it's dead skin coming off. Just things that you're going to see in practice and you're not going to understand when you should have been educated on it. (R#17)

Gaps in nursing education were linked to inadequate community-oriented care. Particularly, participants expressed concern with gaps in community nursing and exposure to diverse patient populations in community settings. This sentiment was linked to non-Black nurses being uncomfortable with visiting ABNS communities for postpartum follow up or homecare. Participants felt education on how social and structural aspects of community are tied to health was necessary.

“It's so important to get a good fix on what's happening in the communities. Where a person lives, what food they are eating, do they have housing? People go into hospitals because of all those reasons” (R#6).

Another participant continued:

“There needs to be more emphasis around community. Exposure activities where people get into different communities and environments. Because it's a different reality to see other communities and how to support people” (R#9).

Participants described professional development, clinical training, and health education as focused largely on disease management rather than preventative strategies and cultural competence. To circumvent this, participants engaged in community-based care including health programs, teaching, and homecare.

#### Subtheme 3: community-oriented care within a medical-based model: providing the best care

All participants spoke about the importance of community-oriented care. Community-oriented care was juxtaposed against a medical-based model, which was described as reactive, disjointed, treatment-focused, with an overuse of medications and interventions. The system was accused of being disconnected from patients, families, and communities, and not empowering patients nor promoting health. Some participants felt conflicted with the medical-based model and questioned their role in the health system.

It's a broken system that's not meant to be fixed. You’re taught medicine, drugs, treatments. It's like, well, now you know how to solve the problem. No, we know how to manage it, not solve it… There's no investigation because they’re not here for that… It just seems like you’re a pill pusher. (R#15)

Another participant described balancing the best interests of the patient with collegial dynamics. This created tension with colleagues when there was a difference in perspective and a desire to go beyond basic care.

As long as you don't go against the grain, you're golden…when you do, it puts a target on your back because you're labeled as a troublemaker. Then you become ostracized. But they can ostracize me all they want because I have a duty to keep these patients safe. (R#8)

For participants, community-oriented care included holistic, patient-centered care, that addressed underlying issues and the social and structural determinants of health, as well as accessible services in the community such as homecare.

Black people work too hard. They have these little houses. We didn’t have mortgages. We had to scrape. People worked hard to have a roof over their head. They're not leaving home. So why don't you put resources in place that keep them in their house, and hire people from the community? People used to say to me, “If you weren’t Black, we wouldn’t let you in here. (R#2)

My heart was in community nursing. I wanted to work in a place where I could support a population that looked like me and had [experiences] that I could relate to. People where I come from, from low income neighbourhoods, fall through the cracks. We don't get the same level of care as affluent populations. (R#10)

Health literacy, illness management, and fostering an openness to discuss health issues, were components of community-oriented care. For example, fostering trust through open dialogue promoted the disclosure of sensitive health information concerning diabetes, obesity, breast cancer, substance dependency, and mental illness. Participants felt it was important for the community to know how to care for themselves and each other, rather than relying on the health system.

I wouldn't look to acute care for the change. It would be community nursing. We have to educate our people. You see them come in…There's no teaching. That's an issue that every race faces. We have this issue with our Black people not knowing how to manage themselves but also we have an issue with everyone else not knowing how to manage themselves. (R#15)

Finally, the importance of spirituality was woven into participant's reflections on how they cared for their patients. Spirituality was poignant at end-of-life, with participants praying with patients and playing gospel music for a peaceful death.

“I say we're like God's angels on earth. Making people comfortable and allowing them to have a dignified, peaceful death. It's rewarding. More than any money I could receive” (R#10).

*Institutions of Care* capture the experiences of ABNS nurses in nursing and more broadly in healthcare. Further, it provides a critical understanding of how ABNS nurses navigate these institutions, while maintaining commitment to culturally appropriate community-oriented care.

## Discussion

This paper presents the findings from Section Two: *Institutions of Care,* which is part of a larger qualitative study that examined the leadership experiences of ABNSs in healthcare. This paper expands upon the foundation established in Section One: *ABNS as a Distinct People* while serving as a precursor to Section Three: *Leadership Philosophy and Practice* (Jefferies et al., 2022a). *Institutions of Care* presents two conceptual themes, ‘*Black Tax’ in Nursing* and *Nova Scotia Healthcare as an Archaic Institution*, which describe the complexity of experiences of ABNS nurses within the nursing profession and the healthcare system. While this paper does not present an exhaustive interrogation of these institutions, it addresses the paucity of evidence related to ABNS nurses and their experiences in healthcare. Importantly, examining the leadership experiences of ABNS nurses revealed opportunities to enhance equity and inclusivity in nursing and the health system.

The first theme, ‘*Black Tax’ in Nursing,* situates the nursing profession as an institution of care, and portrays the process of traversing professional tensions, navigating the health system, and integrating into an evolving profession as an ABNS nurse (Jefferies et al., 2022a). There are common obstacles that all nurses endure, including completing an accredited nursing program, a competency examination, the hiring process, and day-to-day work. This study corroborates evidence regarding the challenges of integrating into the nursing profession, and how this process is further complicated by racism, sexism, and classism (Beagan et al., 2023; Collins, 2004; Cooper Brathwaite et al., 2022; Etowa et al., 2009; Kihika, 2024). The added physical, mental, emotional, and spiritual strain – characteristic of Black tax – was apparent in participants’ descriptions of daily microaggressions as well as systemic processes. Similar experiences, including racist humor, name calling, and surveillance, were identified by Black nurses in Canada (Modibo, 2004; Cooper Brathwaite et al., 2022; Prendergast, 2014; Stewart, 2009). Black Tax was also present in intra-professional dynamics, such as when challenging assumptions related to knowledge and beliefs about leadership and caregiving. Notably, integrating into nursing was equated to an exclusive club with access denied for those deemed “less suitable” or with members admitted through invitation after careful scrutiny. Prendergast (2014) found a similar phenomenon with the “ideal type” in nursing, where all nurses were expected to reflect Victorian ideals and aspects of womanhood (De Sousa et al., 2024; Flynn, 2011; Jefferies & Price, 2021).

The second theme focused on the health system in Nova Scotia – as an institution of care – and how performative practices or tokenism diminished a sense of belonging or reinforced exclusion. Participants identified systemic issues such as diversity gaps in health practitioners, prejudice and harmful stereotypes in education or policies, as well as a profound disconnect between the health system, patients, and providers. Similar health system issues are attributed to racism and colonialism (Boyer, 2017; Mahabir et al., 2021). Participants emphasized the need for culturally appropriate community-oriented health services, which was discussed alongside solutions to address competency gaps, substandard care, and patient harm in nursing. These findings align with Dryden and Nnorom's (2021) recommendation for an interdisciplinary community-informed approach to address racism in Canadian healthcare.

Each of these conceptual themes underscores the historical foundation and legacy of anti-Black racism and colonialism in nursing and healthcare. The recognition and understanding of how these institutions influence and shape contemporary practices – combined with the commitment to systemic transformation – is a precursor to shift nursing and healthcare towards culturally appropriate, high quality, and socially just systems (De Sousa et al., 2024; Flynn, 2011; Jefferies et al., 2022a, 2024; Jefferies & Price, 2021; Philbert et al., 2024).

### Recommendations

The following recommendations were developed by the first author through the process of data analysis. These recommendations focus on systemic and institutional processes to address issues in nursing and the health system. The integration process, in Figure 2, is a useful visual that illustrates opportunities to repair multi-level issues in nursing and the health system. Sustained transformation in nursing and healthcare requires addressing each of the three levels (gatekeepers, policies, and structural) as single components and as a larger system. The first recommendation is for institutions and organizations to perform process audits, including examining institution and organization policies, practices, and positions, to identify issues. This audit will uncover solutions to issues posed by gatekeepers, policies, and structures. Examples of this may include reviewing the current reporting mechanism for anti-Black discrimination in the clinical setting then developing or refining a procedure to address anti-Black racism encountered by nurses or nursing students in the clinical setting. Another example includes reviewing nursing curricula to remove prejudicial or stereotypical content.

The second recommendation is for entities, such as Nova Scotia Health (NSH), the province-wide health authority, to maintain current efforts towards attaining a truly diverse, equitable, and inclusive workforce and health system. NSH has made significant advances by partnering with community organizations and practitioners to launch community-oriented programs, improve access to services, and support innovative community-oriented initiatives (NSH, 2021). Expanding current partnerships and exploring novel collaborations is another step to facilitate the integration of community-specific knowledge and expertise of ABNS nurses into the health system.

### Implications for nursing

This study has implications for nursing in the following areas. For education and practice, this study highlights opportunities to improve nursing curricula as well as opportunities to strengthen program recruitment, retention, and representation. For research, this study reveals the need for quantitative, mixed methods, and evaluation studies in nursing, to collect statistical data and evaluate programs or initiatives. For administration, this study illustrates the issues posed by gatekeepers as well as opportunities to create an inclusive environment and foster a sense of belonging. Finally, the recommendations and findings provide policy considerations for integrating diverse perspectives, ideas, and practitioners from groups that have been historically marginalized and excluded, into healthcare.

### Limitations

The limitations in this study include the use of a single telephone interview during the initial wave of the COVID-19 pandemic. Additionally, the semi-structured interview guide was not pilot tested. Also, triangulation techniques, such as member-checking, were not performed. Finally, this study did not collect any demographic data, which impacts the transferability of the findings.

## Conclusions

This research offers insight into the experiences of ABNS nurses in the nursing profession and health system. By examining the leadership experiences of ABNS nurses, this study offers evidence-informed recommendations to address anti-Black racism in nursing. Transforming nursing and healthcare requires addressing examining issues posed by gatekeepers and antiquated policies or processes, facilitating the recruitment, retention, and successful integration of ABNS nurses. Importantly, this research contributes to addressing anti-Black racism in nursing and healthcare by illustrating a bi-directional multilevel process. *Institutions of Care* provides an understanding for how nursing and the health system influence ABNS nurses’ leadership and how these institutions have implications for the health and wellbeing of individuals, families and communities.
